# Herbal Products in Italy: The Thin Line between Phytotherapy, Nutrition and Parapharmaceuticals; A Normative Overview of the Fastest Growing Market in Europe

**DOI:** 10.3390/ph9040065

**Published:** 2016-10-29

**Authors:** Marco Biagi, Rita Pecorari, Giovanni Appendino, Elisabetta Miraldi, Anna Rosa Magnano, Paolo Governa, Giulia Cettolin, Daniela Giachetti

**Affiliations:** 1 Department of Physical Sciences, Earth and Environment, University of Siena, Via Laterina 8, 53100 Siena, Italy; elisabetta.miraldi@unisi.it (E.M.); annarosa.magnano@unisi.it (A.R.M.); paolo.governa@unisi.it (P.G.); giulia.cettolin90@gmail.com (G.C.); daniela.giachetti@unisi.it (D.G.); 2 Italian Society of Phytotherapy, Via Laterina 8, 53100 Siena, Italy; ritapecorari@ritapecorari.it (R.P.); giovanni.appendino@uniupo.it (G.A.); 3 Department of Pharmaceutical Sciences, University of Eastern Piedmont, Largo Donegani 2, 28100 Novara, Italy

**Keywords:** herbal medicinal products, food supplements, botanicals, normative, phytotherapy, Italian pharmaceutical market, parapharmaceuticals

## Abstract

The Italian herbal products market is the most prosperous in Europe. The proof is represented by the use of these products in several marketing categories, ranging from medicine to nutrition and cosmetics. Market and legislation in Italy are at the same time cause and consequence of this peculiar situation. In fact, the legislation on botanical food supplements in Italy is very permissive and at the same time the market shows an overall satisfaction of users and strong feedback in terms of consumption, which brings a widening use of medicinal plants, formerly the prerogative of pharmaceuticals, to other fields such as nutrition. This review summarizes the market and normative panorama of herbal products in Italy, highlighting the blurred boundaries of health indications, marketing authorizations and quality controls between herbal medicines and non pharmaceutical products, such as food supplements, cosmetics and other herbal-based “parapharmaceuticals”.

## 1. Herbal Products in Italy: Overview of the Market

The Italian term “fitoterapico”, properly translated as “herbal drug” or “herbal medicine” or “phytotherapic medicine” or “plant medicine”, up to twenty years ago strictly referred to drugs obtained by means of extraction or other chemical or physical procedures applied to medicinal plants [1]; being registered drugs, herbal medicinal products were all listed in the official pharmacopoeias of different countries or in reference texts such as World Health Organization Monographs on Medicinal Plants (volume 1 to 4) [2,3,4,5], European Scientific Cooperative On Phytotherapy (ESCOP) Monographs [6,7] or monographs of deutsche Kommission E (Commission E) [8]. Efficacy, safety and chemical standardization of herbal medicines were guaranteed and quality controls well defined. In Italy, during the XIV legislature in 2002, the House of Representatives proposed a law that uniquely considered the definition, the scope, the registration process and the quality requirements of herbal medicines [9]. Since the late 1990s, however, a large number of herbal products, as cut or powdered plants, extracts or other preparations, collectively defined as botanicals, entered into the market also as food, cosmetics and as parapharmaceuticals, term that can be used to define non pharmaceuticals products having health benefits, such as food supplements and novel foods. Due to the increased interest, Europe started to regulate this sector. In 1997, a regulation was issued on “Novel Food” (Regulation 258/97/CE) [10]; then, the community directive which regulates the requirements for the marketing authorization of food supplements (Directive 2002/46/CE) [11] tried to harmonize the lists of "botanicals" used in the food industry, but with little success because of the differences in norms between the different European countries that make it difficult to distinguish between herbal drugs and botanicals foods supplements.

As reported by Silano and colleagues, in Europe, products containing the same botanical source may belong to different marketing categories in the Member States, on the basis of different national approaches, thus confirming the need for an harmonization of the current regulatory framework concerning herbal products [12].

In Italy, the last Management Order of the Italian Ministry of Health of 14 March 2014 [13], which updated the ministerial decree of 9 July 2012 [14], defines the botanicals that can currently be included in food supplements.

Currently, in Italy, the number of botanicals in food supplements is very large.

Supply and demand led to a rediscovered interest from a large segment of the population towards herbal products.

Herbal drugs, as drugs in general, in Italy have fluctuating market trends, stable on average in the last ten years, with a positive trend in the 2015–2016 period [15]. Despite this, all herbal drugs sales are very limited and no plant products are among the top sellers.

On the other hand, the Italian market is the most flourishing in the European scene both concerning nutritional supplementation overall (over 2.5 billion euro in the period May 2015–May 2016, [16]) and concerning botanical food supplements (a little less than 1 billion euro in the period May 2015–May 2016, [16]). In Italy, the botanical food supplements market is worth more than 4% of the overall revenue produced by the pharmacy sale channel (25.4 billion euro in the period May 2015–May 2016, [16]) stably in the first place for the sale of drugs and parapharmaceuticals. The botanical food supplements market is currently worth four times that of homeopathy and three times that of veterinary drugs [16].

In Italy, it is possible to associate with the botanical food supplement a health claim that recognizes a biological function, whether it be prevention, maintenance of homeostasis, or normalization of biological parameters [17].

Other European countries are looking to harmonize these claims but also in this case with great difficulty; the European Food Safety Authority (EFSA) today recognizes very few claims associated with plant derivatives and these only refer to active ingredients or classes of active ingredients of medicinal plants in well-defined dosages (for example, 5 mg/*die* of olive oil polyphenols expressed as hydroxytyrosol to avoid lipoproteins peroxidation or 10 mg/*die* of monacolin from red yeast rice to maintain regular blood cholesterol levels) and not, as in Italy, to the botanicals [18]. The number of botanicals allowed in Italy as ingredients in food supplements is very large and to date includes two lists: one, the Italian list, and another, the so-called BELFRIT (BELgium FRance ITaly) list resulting from a collaboration between Belgium, France and Italy. Every European country defines its own list of plants usable in food supplements and soon also the Italian Ministry of Health will define only one list that will include most of the plants placed on the two lists.

Taking into account that food supplement marketing authorisation is impressively cheaper and easier compared to that of herbal medicines, it is no surprise that the largest part of medicinal plants in Italy are used as botanical food supplements, associated with health claims ranging from the regularization of the intestinal transit, the maintenance of the immune system, to the normalization of cholesterol levels [17,18], to only name the top-selling products, which makes it difficult to draw the line between therapeutic and healthy functions; currently, despite a clear legal definition, the meaning of the Italian term “fitoterapico” (phytotherapeutical) is conflicting. The Ministry of Health, in the section dedicated to "Fitoterapici", still defines them as "herbal medicines" distinguishing them, in the same ministerial note of 17 January 2013 [19], from the so-called “Prodotto di erboristeria”.

Market and legislation in Italy are at the same time cause and consequence of this peculiar situation; it is true that the legislation on botanical food supplements in Italy is one of the most permissive ones in Europe, which can only be compared to Spain, for the use of botanicals in food supplements, and, at the same time, the market has given an impressively positive feedback in terms of uniqueness of consumption in Europe, which helped to widen the use of medicinal plants in the food field.

Part of this success could undoubtedly also be due to the fact that family physicians have controlled budgets relating to prescription drugs that leads to prescriptions of botanical food supplements, paid by patients, in the case of mild forms of chronic diseases and supplements, instead of drugs reimbursed by the national Health Service. As medicinal plants taken orally are currently also used in food supplements, those that have topical use are also interestingly utilized in functional cosmetics, respecting the competence of regulation and purpose of use of this products.

The Italian and European scenario of the use of herbal products is hence very complex, and we find that, over medicines, food supplements and cosmetics, botanicals could be marketed also as food, for example spices or herbal teas, or as foods for special medical purposes (recently governed by EU Regulation 128/2016) [20], or as novel foods (EC Regulation 258/97) [10], which identify products intended to be consumed as food that have no sufficient tradition of Community-wide use, or, finally, as Medical Devices (Law Decree No. 46 of 24/02/1997) [21], when the plant product can demonstrate a sole mechanical and non-pharmacological action, such as protection of mucous membranes or skin cooling or warming effect.

Labeling, marketing authorization, purpose of use, quality control of herbal products are linked to the corresponding product category.

Below the normative relative to drugs, to pharmacy preparations (also defined as galenic formulations), to food supplements and cosmetics, covering over 95% of the market for herbal products in Italy, is reported.

## 2. Regulation of Herbal Products Marketed in Italy

### 2.1. Drugs

Article 1 of D. Lgs. 24 April 2006, n. 219 defines herbal drug or herbal medicine (it. fitoterapico) as a medicine containing one or more herbal substances or one or more herbal preparations or one or more herbal substances in association with one or more herbal preparations as active principles [22]. Herbal substances encompass the whole plants, parts of plants, algae, fungi, lichens, as a whole, in pieces or cut, non-treated, usually desiccated but sometimes fresh, and some exudates that do not undergo specific treatments, precisely defined on the base of the plant part used and its botanical name, according to the binomial nomenclature (genus, species, variety and author). Herbal preparations include preparations obtained from herbal substances using treatments such as grinding, pulverization, mechanical extraction, extraction with solvents, distillation, expression, splitting up, purification, concentration or fermentation and consist of grinded or pulverized drugs, tinctures, extracts, essential oils, juices from squeezing out and processed exudates.

In general, a medicine can either be industrially produced or prepared by pharmacists in drugstores.

In the European Pharmacopoeia 8th ed. and in the EC national pharmacopoeias, quality control methods and requirements of herbal medicines are reported. Both raw materials and end products are submitted to quality control, especially to the ones concerning the exact botanical characterisation through pharmacognostic methods contained in the pharmacopoeias, the control of organic and chemical contaminants, the control of fertilizer traces, heavy metals, microbial load and aflatoxins, radioactive substances. The book “Quality control methods for medicinal plant materials” published by World Health Organization (WHO) also reports in detail the quality requirements of herbal medicines [23] while the “WHO Guidelines on Good Agricultural and Collection Practices (GACP) for Medicinal Plants” is a technical guidance on obtaining medicinal plant materials and herbal products of good quality classified as medicines [24]. Pharmacopoeias and official reference texts unequivocally establish drug preparation procedures and herbal medicine chemical requirements, specifying for each product the active principles content and the analysis methods.

#### 2.1.1. Industrial Drugs

Drugs of industrial origin must be authorized by the Italian Drugs Agency (AIFA) or by the European Medicines Agency (EMA), which verify their quality, efficacy and safety, and they can only be sold in drugstores.

Herbal products possessing a history of therapeutic use of at least ten years within the European Community, with a recognised efficacy and an acceptable level of safety, are defined as well-established use, and their marketing authorisation is regulated by article 10a of Directive 2001/83/EC [25], which also establishes the allowed routes of administration including the oral, the parenteral, the topical and the inhalation routes. Some examples of well-established use herbal products most sold in Italy are: Permixon^®^ (*Serenoa repens* (Bertram) Small fruits), Legalon^®^ (*Silybum marianum* Gaertn. Fruits), Nervaxon^®^ (*Hypericum perforatum* L. flowering tops), Centellase^®^ (*Centella asiatica* L. Urban purified triterpenic fraction), Daflon^®^ (micronized purified flavonoic fraction derived *Citrus* spp.), and Tegens^®^ (*Vaccinium myrtillus* L. fruits).

Timing and costs of marketing authorisation for well-established herbal medicinal products are the same for every other drugs, consisting in more than ten years for preclinical and clinical trials and millions euros of overall expenses.

If the active substances of a herbal product have been used in medicine for at least 30 years, of which at least 15 within the EU, even if lacking the support from pre-clinical and clinical trials, it is possible to submit the drug to the so-called traditional use registration.

The simplified procedure is contained in the Directive 2004/24/EC, amending, as regards traditional herbal medicinal products (THMP), Directive 2001/83/EC on the Community code relating to medicinal products for human use [24], along with the allowed routes of administration (oral, topic, inhalation). The Committee on Herbal Medicinal Products (HMPC) of EMA gathers in monographs scientific opinions on herbal substances and preparations, with information on recommended uses and safe conditions, thus encouraging companies who seek a traditional use registration for their product. EMA has published 184 community herbal monographs that contains one or more traditional preparations. Despite this, only three THMPs are currently available in Italy; *Pelargonium sidoides* DC. (Kaloba^®^) roots standardized extract was the first THMP registered and it is still the most sold in Italy. Even though quality controls and manufacturing procedures required for THMP remain the same as other herbal medicines, the simplified procedure for marketing authorisation does not oblige to perform conventional experimental procedures, yet reviewed and considered in HMPC community monographs. Directive 2004/24/EC implies a remarkable reduction in terms of costs, which is different for each European country. THMP authorisation in Italy is relatively almost as expensive, but limited to some tens of thousands euros.

#### 2.1.2. Galenic Formulations

Italian law allows pharmacists to set up galenic formulations in the laboratory drugstore. These are drugs prepared directly by pharmacists, and they are intended to obtain formulations and/or drugs dosages not available as registered drugs.

Galenic formulations consist of magistral and officinal preparations.

Article 3 of D. Lgs. 24 April 2006, n. 219 [22] states that the first category includes drugs prepared extemporarily on the basis of a medical prescription to a given patient and is disciplined by article 5 of D. L. 17 February 1998, n. 23 [26], successively converted with some modifications by L. 94/98 [27], and the second contains drugs, their preparations or drug association enlisted in European Pharmacopoeia (Ph. Eur.), national pharmacopoeias in force in the EU member states or officinal formularies and prepared according to appropriate reference texts (i.e., pharmacopoeias). Officinal preparations are designed to be provided directly to the patients served by that drugstore.

Officinal preparations have always to respect the Italian Pharmacopoeia (FUI) in some aspects. Contrarily to magistral preparations, officinal ones can be set up whether they require a medical prescription or not, even in advance but in limited quantity, provided that stocks do not exceed 3 kg per formula. Every single batch needs to be marked with a number corresponding to its worksheet.

L. 94/98 enables physicians to prescribe magistral preparations having as active principles any existing active substance described in the pharmacopoeia of an EU member state, as well as any active substance contained in industrially produced medicines already authorized either in Italy or in another EU member state, or with a revoked or unconfirmed marketing authorisation (AIC). In magistral preparations, they can also employ any existing active substance contained in non-medicinal products or cosmetics properly commercialized in the EU as active principles, obviously, in the first case for oral administration and in the second for topical application. In order to safeguard public health, the Ministry of Heath may impose bans and restrictions to magistral prescriptions. In the case of magistral preparations with different indications from industrial product analogous, physicians have to demonstrate the legitimacy of resorting to that specific extemporaneous preparation, provide documentary evidence of the informed patient consent obtained, and indicate the alphanumeric code uniquely identifying the patient in the prescription, in place of his or her name and surname. Original or copies of the prescriptions received by pharmacists, whether repeatable or unrepeatable (Tables 4 and 5 of the Italian Pharmacopoeia), have to be sent every month to Local Health Authority (ASL), which, in turn, sends them to the Ministry of Health.

Preparations are based on following the medical prescriptions, with monographs and rules for proper preparation included in the FUI as references.

Galenic preparations have to be set up by the pharmacist according to Good Preparation Practices and have to fulfill Pharmacopoeial quality requirements.

### 2.2. Food Supplements

Dietary supplements are defined in Directive 2002/46/EC [11], put into effect by D. Lgs. 21 May 2001, n. 169 [28], as dietary products intended to complete common diet and consist of a concentrated source of nutritional substances or other substances that possess a nourishing or physiological effect.

Herbal ingredients (i.e., entire plants or their parts, non-treated and generally desiccated) and herbal preparations (i.e., herbal ingredients that have undergone various treatments, such as extraction, distillation, pressing, splitting up, purification, concentration, fermentation, grinding or pulverization), defined as botanicals, can be included in dietary supplements.

In Italy, D. M. 9 July 2012 [14] and its update, D. M. 27 March 2014 [13], discipline the use of herbal ingredients and preparations in dietary supplements. D. M. 9 July 2012 supports and integrates D. Lgs. 21 May 2004, n. 169, fulfillment of Directive 2002/46/EC, which provides indications for harmonization of vitamins and minerals only, letting herbal products be governed by national legislation of each member state and by the mutual recognition principle. D. M. 27 March 2014 adds to a preceding national list of about 1200 botanicals approved in dietary supplements (attachment 1 of D. M. 9 July) the so-called BELFRIT list (attachment 1 bis of D. M. 27 March 2014), continuously being updated and containing by itself approximately 1000 botanicals of which more than 120 are “new” for Italy [13]. The project BELFRIT, which started on 18 April 2013 during the conference “Botanicals in Food Supplements” held in Rome, starts with the definition of a harmonized list of admissible plants in food supplements, sustained by current scientific evidence, and represents the first stage in order to establish common rules, shared between European countries. Attachment 1 of D. M. 9 July, intended to be fully replaced by the BELFRIT list in the future, reports reference indications for physiological effects (i.e., claims) referring to the botanicals included and determined by ministerial guidelines. Pursuant to and by effect of Regulation (EC) n. 178/2002 [29], the Unique Commission for Diet and Nutrition developed the Guidelines on documents supporting the use of herbal substances and preparations in dietary supplements. The guidelines provide information about necessary documents and controls for safety of use of those ingredients.

Bud preparations of officinal plants, well known and used in many European countries, in Italy are mostly considered as food supplements.

Obviously, due to their pronounced pharmacological effect, some medicinal plants are not included on the national or on the BELFRIT list (e.g., *Digitalis* spp., *Ephedra* spp., *Taxus baccata* L. and *Artemisia annua* L.), whereas others can be used only when their when the daily intake of their active principles is kept under strict control, essentially preventing their pharmacological action (for example, maximum daily dose of 0.7 mg hypericin from *Hypericum perforatum* L., with a hyperphorin:hypericin ratio of less than 7, maximum daily dose of 96 mg salicylates from preparations of *Salix* spp., maximum daily dose of 30 mg synephrine from *Citrus* x *aurantium* subsp. *amara* (Link) Engl.).

Quality control of botanical food supplements is definitely different from one of the drugs and this law is based on the Italian Ministry of Health regulations.

Quality control mandatory for botanicals used as food supplements features in particular: heavy metals such as lead, mercury and cadmium and arsenic, microbial load and aflatoxins, pesticides, residual solvents and radioactivity. Heavy metals and arsenic limits in botanical food supplements are defined by the Reg. (EC) 629/08 [30] and by the self-regulation document issued by Italian Category Associations operating on botanicals: Codex Herbarum [31]. The limits are set to be very similar to those of herbal medicines and more rigorous regarding mercury (0.3 ppm in herbal medicines and 0.1 ppm in botanical food supplements). Microbial load limits vary for different botanicals (i.e., powders, powders to be used in boiling water, extracts) and consider total viable bacteria, total yeasts count and biliar salts resistant Gram-bacterial count. *Salmonella spp*., *Escherichia coli* and *Staphylococcus aureus* must be absent in every botanical food supplement. Limits for pesticides and aflatoxins are quite similar, even if more permissive, of those of herbal medicines. In the food field, limits of residual solvents are more restrictive than the ones for pharmaceuticals and only food-grade solvents as water, glycerine, ethanol and, with well-defined limits, ethyl acetate is admitted in product preparation.

In the case of a regulations gap, most references to raw materials and end products are taken or adjusted from pharmacopoeias and assimilated to that of herbal medicines.

Moreover, dietary supplements require their nutritional values be included on the label, their recommended daily intake and the dosage for each posological form. Within the limits explained above, the regulation of dietary supplements does not impose specific preparation techniques allowing for obtaining plant extracts and herbal preparations; it does not require producers to quantify the active principles (except in the case of pre-established daily intake limits, e.g., hypericin or synephrine) and, in any event, it does not force them to use certain methods of analysis for botanical standardisation either. The food supplement marketing authorisation request in Italy is very cheap, costing 160.20 euros.

### 2.3. Herbal Preparations

In Europe, a legal definition of the Italian term “prodotto erboristico” (herbal products) does not exist, while the Italian definition of herbal products indicates herbal preparations intended to be only sold to customers and exclusively packaged at the point of sales of pharmacies and parapharmacies or in the herbalist’s shops, in the last two cases only in the presence of food laboratories authorized by local health units. Plant preparations and derivatives can be commercialized as herbal products in accordance with the Italian Ministry of Health notes n. 600.12/AG45.1/706 of 5 December 2002 and DGSAN 0015807-P-19/05/2010 of 19 May 2010 [32,33]. The set-up products, considered as unpackaged, are equated to foods and therefore they can only be assumed orally, cannot boast pharmacological properties (drugs) or health benefits (dietary supplements) and can contain exclusively botanical species accepted to be used in foods, thus both plants are part of the diet and the ones included on the national and the BELFRIT lists. Herbal products can be extemporarily prepared or stored but within the limits established for officinal preparations, up to 3 kg.

Pharmacists set up herbal preparations according to Good Preparation Practices and to the national legislation on food preparation, with reference to products prepared in parapharmacies and herbalist’s shops; quality requirements of herbal products usable to prepare herbal preparations remain the same as those of packaged botanical food supplements.

### 2.4. Cosmetics

Any substance or mixture intended to be placed in contact with the various external parts of the human body (epidermis, hair, nails, lips and external genital organs) or with the teeth and the mucous membranes of the oral cavity, with a view exclusively or mainly to cleaning them, perfuming them, changing their appearance and/or correcting body odours and/or protecting them or keeping them in good condition. Regulation (EC) n. 1223/2009 does not only give the definition of “cosmetic product”, and it contains a series of rules ensuring the safety of cosmetics from different points of view (from the manufacture method to the ingredient control, to the mandatory labelling and the expert evaluation) [34].

Since the regulation is enforceable as law in all member states, it establishes that some of the information on the label, such as purpose, warnings and instructions for use, have to be reported in the official language of the country where the product is sold.

Plant products in cosmetics, as well as in dietary supplements, cannot be used with a therapeutic aim, but with the sole purpose of maintaining skin integrity.

The inclusion of plant products in cosmetics does not imply variations in quality controls and requirements of those products, thus the reference legislation is the one established by Regulation (EC) n. 1223/2009 [34].

As in the case of food supplements, the marketing authorisation request for cosmetics is cheap and affordable for companies working with herbal products.

## 3. Discussion

Italy has a long tradition and knowledge in the use of herbal products, and it is currently the European country with the fastest growing market, since they are used as food, novel foods, food supplements and cosmetics when intended to maintain health more than as herbal medicines with therapeutic purposes.

Among the more than 50,000 herbal products available, people usually choose the ones that better respond to their health needs, often ignoring the fact that diverse marketing categories imply deep differences in terms of manufacturing processes, chemical composition, quality controls, studies of efficacy and price.

The marketing authorization of herbal medicines entail the assessment of therapeutic efficacy with preclinical and clinical studies, and the accomplishment of Good Preparation Practices and the distribution mainly through pharmacies, under the control of an expert who can recommend their use.

All registered herbal medicines have strong scientific data support. For example, more than 150 clinical trials reporting the hepato-protective action of sylimarine from *Sylibum marianum* Gaertn. fruits (Legalon^®^) are published in Scopus and PubMed databases; more than 60 clinical studies, instead, regard the standardized lipidosterolic extract of *Serenoa repens* (Bartram) Small fruits (Permixon^®^), used for benign prostatic hyperplasia.

In addition, THMPs are well studied and over 20 clinical studies have been recorded on the standardized extract of *Pelargonium sidoides* DC. roots used in the case of cold symptoms.

A recent review by Minghetti and colleagues suggested the need for a simplified pharmaceutical development procedure for herbal medicinal products with a certain grade of innovation (i.e., proposed for new indications), in order to increase the number of safe and effective products on the market and to improve the therapeutic index of traditionally utilized products [35].

On the other hand, foods and food supplements only require the obligation of notification to the Ministry of Health that ensure safety of use, while efficacy is only referred to the use of allowed substances with health claims. Hazard Analysis and Critical Control Points (HACCP) procedures are required to manufacture these products. In addition, efficacy is not to be proven for cosmetics.

It is important to bear in mind that, beyond being certainly easier, marketing a food supplement is definitely cheaper than marketing an herbal medicine. This is likely to make the food supplements market completely overwhelm the one for herbal medicines.

The quality standards in botanical food supplements vary from one product to another. Companies are beginning to significantly improve their quality standards, often proposing extracts and formulations increasingly similar to herbal medicines. It is even possible that herbal drugs, marketed abroad as herbal medicine, in Italy are registered only as botanical food supplements. The most representative cases are those of standardized extracts of *Panax ginseng* CA. Mayer roots and *Gingko biloba* L. leaves. Obviously, the quality and the effectiveness of these “botanicals” is well documented. Nevertheless, to avoid safety concerns and maintain the price, often botanical food supplements containing pharmaceutical grade extracts are significantly underdosed.

Unfortunately, this notable attention to the quality still represents the smallest percentage of botanical food supplements available in Italy, and more than 90% of ingredients and formulations sold as botanical food supplements have not undergone clinical investigations.

In this context, it is evident that physicians and pharmacists, above all, have the correct and rational use of parapharmaceuticals on hand, orienting users towards the choice of high quality products that could actually be safe and effective.

Magistral and officinal galenic preparations guarantee the quality and often represent the best choice among herbal products; nevertheless, these preparations are not often used because people scarcely know this category of products, physicians are not often adequately trained to prescribe herbal products and pharmacists having the opportunity to prepare galenics are limited in number.

## 4. Conclusions

Despite an ambiguous legislative situation that still requires a lot of work to be done, the scenario concerning herbal products in Italy is currently very fascinating due to the ever-increasing satisfaction of users and the constantly increasing market, which also led to a re-discovered new interest of the international scientific community on medicinal plants. The fundamental step towards the diffusion and spread of the use of herbal products has been initiated, in the future perspective of a more scientific and rationale use of these products. Undoubtedly, this current phytotherapy is the medical discipline that, being so diverse and multidisciplinary, more than many others, is geared toward modern medicine, focused on the individual, treating not only the symptoms of disease, but also providing benefits for the prevention of disease and health maintenance.
